# A non-pharmacological therapeutic approach in the gut triggers distal metabolic rewiring capable of ameliorating diet-induced dysfunctions encompassed by metabolic syndrome

**DOI:** 10.1038/s41598-020-69469-y

**Published:** 2020-07-31

**Authors:** Carolina Magdalen Greco, Stefano Garetto, Emilie Montellier, Yu Liu, Siwei Chen, Pierre Baldi, Paolo Sassone-Corsi, Jacopo Lucci

**Affiliations:** 1 Department of Biological Chemistry, School of Medicine, Center for Epigenetics and Metabolism, U1233 INSERM, University of California, Irvine (UCI), Irvine, CA 92697 USA; 2 Natural Bio-Medicine SpA, Loc. Aboca 20, 52037 Sansepolcro, AR Italy; 3 Innovation & Medical Science Division, Aboca SpA Società Agricola, Loc. Aboca 20, 52037 Sansepolcro, AR Italy; 4 Department of Computer Science, Institute for Genomics and Bioinformatics, UCI, Irvine, CA 92697 USA

**Keywords:** Physiology, Metabolism, Metabolic diseases, Obesity

## Abstract

Metabolic syndrome has increased at a worrisome level. Lifestyle changes are not sufficient to prevent and improve the adverse effects of obesity, thus novel interventions are necessary. The aim of this study was to investigate the use and metabolic outcomes of a non-pharmacological intervention in a high-fat diet (HFD) fed mouse model, capable of recapitulating key aspects of metabolic syndrome. We show that Policaptil Gel Retard has remarkable, beneficial effects on metabolic dysfunction caused by consumption of HFD. We describe the mechanism by which such effects are obtained, highlighting the fact that the amelioration of metabolic function observed upon Policaptil Gel Retard administration is profound and of systemic nature, despite being originated by sequestering, therefore non-pharmacological events elicited in the gut lumen.

## Introduction

Obesity and its associated comorbidities, such as type 2 diabetes, cardiovascular disease and metabolic syndrome (MetS), are a major public health problem that is increasing at a worrying rate worldwide^1^. Over the last decades, changes in dietary habits have significantly contributed to the rise of metabolic disorders. Indeed, western diets rich in saturated fats and carbohydrates promote metabolic disruption and the development of MetS^2^. Similar to what is observed in humans, feeding mice a high-fat diet (HFD) results in obesity, hyperglycemia, insulin resistance, dyslipidemia and increased levels of free fatty acids in the blood, either independently or concurrently^3^. Hence, high-fat diets have been extensively used to induce obesity and MetS in experimental animals^4,5^.

Animal models of obesity are characterized by extensive changes in the composition and function of the gut microbiota. Several studies have revealed that alterations in the composition of the gut microbiota in response to HFD can affect energy balance and contribute to the onset of obesity and metabolic disorders^6–8^. In addition, dietary interventions as well as therapeutic agents used for metabolic disorders elicit their beneficial effects at least in part through the microbiota. Indeed, many clinical benefits of the anti-diabetic agent Metformin have been linked to gut microbiota dependent effects^9–11^. Thus, the gut microbiota is viewed as an increasingly important target involved in the management of the metabolic complications associated with obesity.

The circadian clock is an endogenous, time-tracking system that directs multiple metabolic and physiological functions required for organism homeostasis. The clock adapts to environmental changes, specifically light–dark cycles, as well as rhythmic food intake^12^. Numerous metabolic pathways, including those involved in glucose and lipid metabolism, display circadian patterns both at the level of gene expression and of intermediate metabolites^13^. Current evidence suggests that disruption of circadian rhythms leads to a number of pathological conditions associated with altered metabolism. Circadian rhythm disruption can be caused by several environmental triggers, among which dietary habits play a crucial role^14^. Mice fed a high-fat diet (HFD) reprogram their hepatic transcriptome by dampening the diurnal expression of numerous circadian targets^15^.

Lifestyle changes, while highly pledged, are not sufficient to prevent and improve the adverse effects of obesity, thus novel interventions are warranted. In the search of alternative interventions, we explored the effect and mode of action of Policaptil Gel Retard, a commercially available product from Aboca SpA Società Agricola. Policaptil Gel Retard is a medical device designed to operate by generating a network of soluble and insoluble fibers typically not absorbed by the body^16,17^ and intended to reduce the absorption of carbohydrates and lipids from the diet, thus reducing the extent and speed of the increase in glycaemia after meals, and leveling out the concentration of glucose and insulin in the blood^14,18,19^. Consistently with the formulation strategy of the product, enhanced swelling and water binding capacities of the network of fibers generated by the medical device was demonstrated^20^. Such features are indeed acknowledged as being related to the ability to physically sequester lipids and carbohydrates from the diet, therefore to the clinical efficacy of the product^16,17^.

Here we therefore study the impact of a non-pharmacological approach on several metabolic parameters dysregulated in a model of diet-induced obesity. We further decipher the mechanisms by which it exerts its effects, linking them to the modifications in the composition of the gut microbiota and to rewiring of time-dependent transcription in the liver.

## Results

### Policaptil gel retard possesses mechanical features known to confer the capability to sequester lipids and sugars

Fibers with both water soluble and insoluble features have been previously described as capable of generating gels that can interact with dietary constituents such as lipids and sugars^21–26^ as they display high water binding (WBC) and swelling capacity (SC). Yet, careful design of specific blends, possibly adding multiple components, can lead to the identification of mixtures with enhanced features. Policaptil Gel Retard, in fact, was already described as a medical device that exhibits water binding capacity (WBC) and swelling capacity (SC) higher than those obtainable by the simple sum of the individual ingredients of the composition (Supplemental Table 1)^20^, thus highlighting a synergistic behavior within the formulation. Policaptil Gel Retard composition is therefore capable of generating a larger gelled and viscous system, considered to confer an improved capability of sequestering lipids and sugars from the diet.

### Policaptil gel retard improves parameters defining metabolic syndrome such as weight gain, insulin sensitivity and serum markers levels in a high-fat diet (HFD) fed mouse model

High-fat diet (HFD) is an extensively used model of metabolic syndrome in mice which effectively promotes obesity, insulin resistance and dyslipidemia^27^. To investigate whether Policaptil Gel Retard could counteract weight gain and insulin resistance in a HFD fed mouse model, we used two experimental protocols. In the first experimental set-up, mice were subjected to a normal diet (ND) or high-fat diet (HFD) for two weeks ad libitum and treated with Policaptil Gel Retard or vehicle starting from day 0 and throughout the two weeks of experiment. Despite no differences in food intake among the experimental groups (Supplementary Fig. 1a), challenge with HFD caused a pronounced body weight increase in HFD control mice, whereas mice treated with the product displayed a full protection against weight gain (Supplementary Fig. 1b,c). We did not observe any changes in body weight between ND and ND-Policaptil Gel Retard (Supplementary Fig. 1b,c). HFD fed C57BL/6 J mice are characterized by marked glucose intolerance and compromised insulin response as early as one week of diet^28^. Thus, we tested if the tested product had a beneficial effect on glucose homeostasis. Policaptil Gel Retard treatment enhanced glucose clearance during oral glucose tolerance test (OGTT) as compared to HFD vehicle control mice (Supplementary Fig. 1d). HFD Policaptil Gel Retard treated mice displayed high peak blood glucose similar to that of control mice (t = 15 and 30 min post glucose bolus). However, blood glucose rapidly reversed to baseline 60 min post glucose bolus, resulting in a significant reduction of AUC, comparable to the AUC of lean controls (Supplementary Fig. 1e).

**Figure 1:**
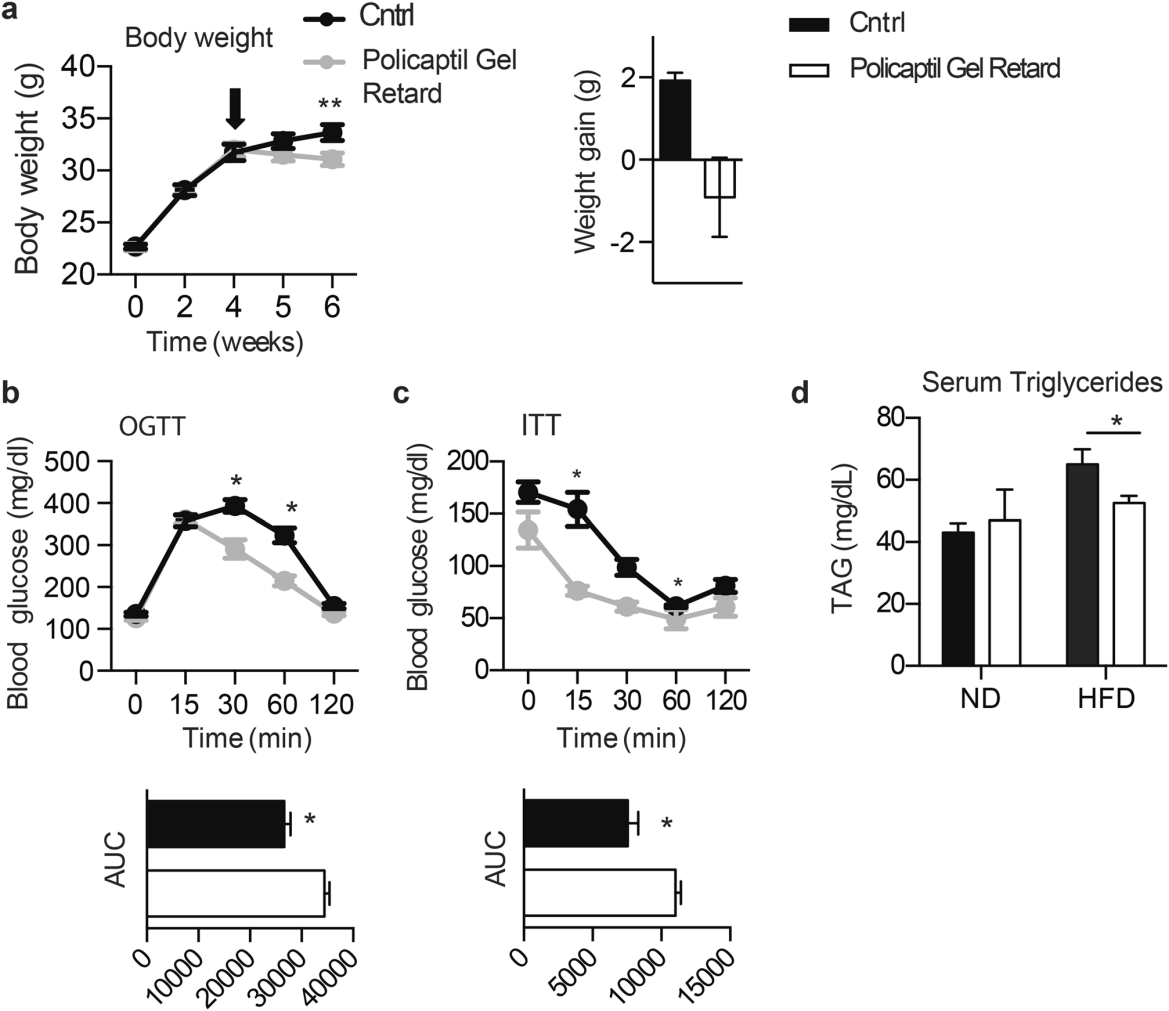
HFD-fed mice treated with Policaptil Gel Retard display improved metabolic parameters. (**a**) **left** Graph of body weight of mice fed HFD and treated with vehicle (HFD Cntrl) or Policaptil Gel Retard (HFD Policaptil Gel Retard) for 6 weeks (mean ± s.e.m, n = 10; **p < 0.01; ANOVA, Bonferroni post hoc). The arrow indicates start of treatment. (**a**) **right** Grams of weight gained/loss after starting treatments (between week 4 and week 6) (n = 10/group). (**b**) Effect of Policaptil Gel Retard on glucose tolerance in HFD-fed mice as measured by oral glucose tolerance test (OGTT) (mean ± s.e.m, n = 5; *p < 0.05; ANOVA, Bonferroni post hoc and unpaired student’s t-test). (**c**) Effect of Policaptil Gel Retard on insulin sensitivity in HFD-fed mice as measured by insulin tolerance test (ITT) (mean ± s.e.m, n = 5; *p < 0.05; ANOVA, Bonferroni post hoc and unpaired student’s t-test). (**d**) Serum concentration of triglycerides (mean ± s.e.m, n = 5; *p < 0.05; ANOVA, Holm-Sidak post hoc).

We then investigated whether Policaptil Gel Retard could arrest or reverse body weight gain in overweight mice. We fed mice HFD for 4 weeks, while another group was fed a normal diet (ND). Both ND and HFD treated mice were randomized into two groups respectively and treated with vehicle or tested product for 2 weeks. During the two weeks of treatment HFD-control animals showed sustained weight gain. Meanwhile HFD mice receiving Policaptil Gel Retard displayed a decrease in body weight, which became more consistent by the second week of treatment (Fig. 1a-left). During the last two weeks of the experiment HFD-control mice displayed a 6% increase in their weight, whereas HFD-Policaptil Gel Retard treated mice lost around 2% of their body weight (Fig. 1a-right). Mice were then tested to examine metabolic parameters that typically define metabolic syndrome in humans. Glucose and insulin tolerance were evaluated by performing a glucose tolerance test (OGTT) and insulin tolerance test (ITT), respectively. Treatment with the tested product markedly improved glucose clearance compared to vehicle diet-induced obese mice (Fig. 1b) and ITT revealed improved insulin sensitivity in Policaptil Gel Retard treated mice (Fig. 1c). Moreover, serum lipid analysis showed that triglyceride (TAG) levels in vehicle treated obese mice were significantly diverging from those seen in normal diet fed mice (Fig. 1d). Notably, Policaptil Gel Retard treatment significantly rescued the TAG serum content (Fig. 1d), demonstrating its beneficial metabolic outcomes.

### Policaptil gel retard induces a specific hepatic signature

**Figure 2:**
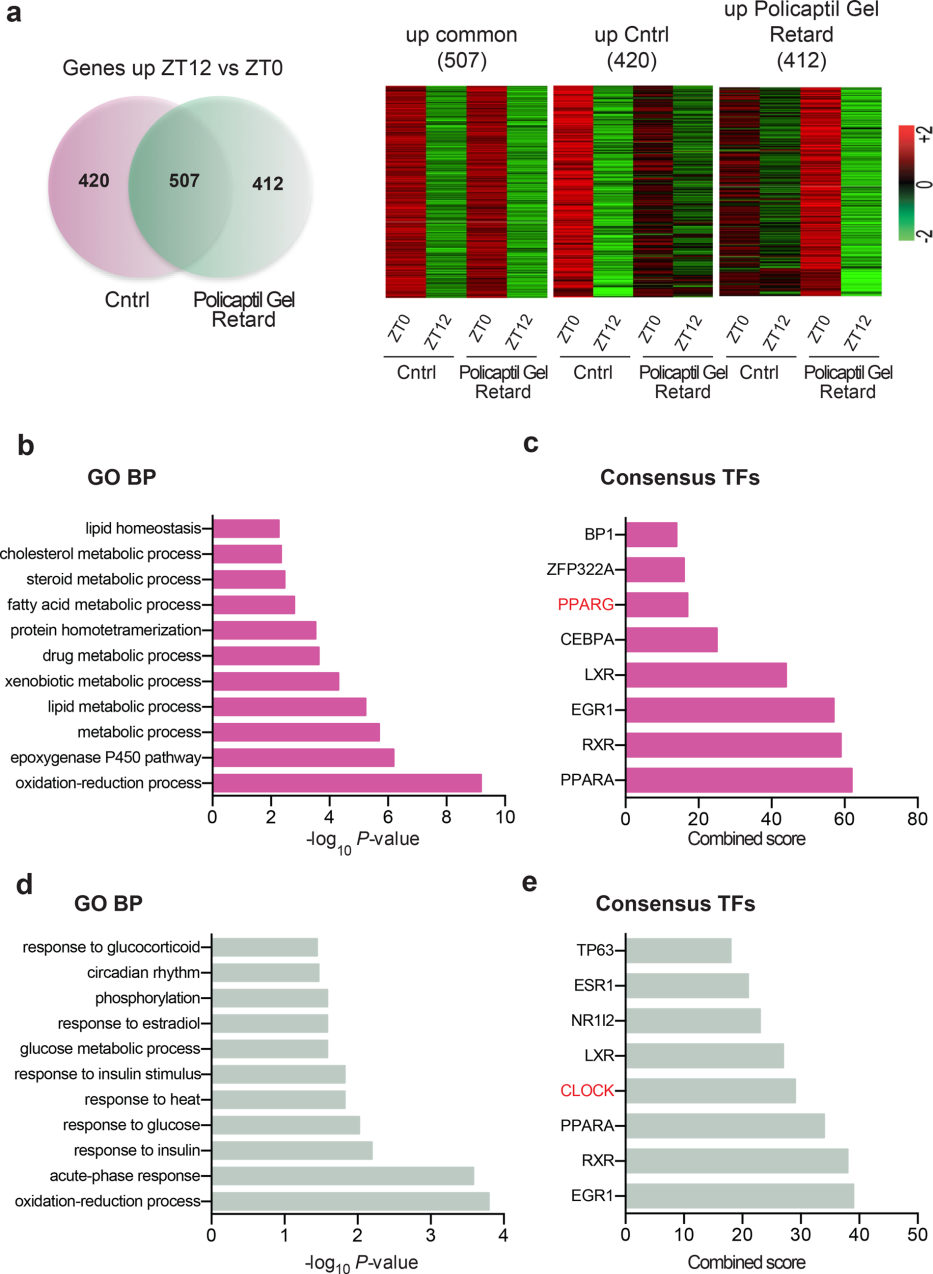
Hepatic transcriptional reprogramming induced by Policaptil Gel Retard. (**a**) **left** Venn diagram of genes whose expression is significantly increased at ZT12 compared to ZT0 in both groups (507), controls only (420) and Policaptil Gel Retard only (412) (n = 3, *Cyber t-test* p < 0.05; ZT Zeitgeber time). (**a**) **right** Heat map of genes significantly up-regulated between ZT0 and ZT12 in vehicle (Cntrl) or Policaptil Gel Retard (Policaptil Gel Retard) treated mice (n = 3, *Cyber t-test* p < 0.05). (**b**) Gene ontology analysis of genes induced at ZT12 in control mice. (**c**) Top 10 results for TF binding sites from ENCODE and ChEA in control mice. (**d**) Gene ontology analysis of genes induced at ZT12 in Policaptil Gel Retard treated mice. (**e**) Top 10 results for TF binding sites from ENCODE and ChEA in Policaptil Gel Retard treated mice.

The liver is the largest metabolic organ of the body and as such integrates numerous metabolic processes, including lipid and glucose metabolism as well as xenobiotic detoxification. Several hepatic metabolic pathways are expressed in a circadian manner and are highly susceptible to nutrient metabolism challenges. In fact, HFD induces extensive metabolic changes in the liver resulting in disrupted circadian metabolic rhythms, insulin resistance, and consequently in the pathogenesis of obesity and Type 2 diabetes^15,29–31^. To explore if Policaptil Gel Retard modulates hepatic gene expression, we performed RNA-seq from livers of mice fed HFD for 6 weeks and receiving the tested product or vehicle for the last two weeks. Samples were collected at two diurnal time points: ZT0 and ZT12 (the end and beginning of the mouse active phase). We first identified genes induced at ZT12, which coincides with the time of day when the mice start to eat, by performing a differential analysis between ZT12 and ZT0 expressed genes. We identified 507 genes significantly increased in both groups, 420 genes induced only in control mice and 412 genes specifically induced in Policaptil Gel Retard treated animals (Fig. 2a). Functional enrichment analysis of genes activated in solely the control group revealed several categories linked to lipid homeostasis and storage, cholesterol and fatty acid metabolism and xenobiotic metabolic process (Fig. 2b). These results are consistent with de novo oscillation of genes related to lipid and fatty acid metabolism in response to HFD^15^. HFD induces gain of rhythmicity of several transcription factors (TF) involved in lipid metabolism, including the peroxisome proliferated activated receptor γ (PPARγ) and the sterol regulatory element-binding protein (SREBP)^15,32^. Indeed, enrichment analysis using EnrichR^33^ with data from ChEA^34^ showed that PPARγ was one of the most represented TF binding genes induced at ZT12 in the control group (Fig. 2c). RT-qPCR confirmed induction of rhythmic expression of the *Pparg* and of its targets DFFA-like effector c (*Cidec)* and truglyceride storage-associated protein (*CD36)* (Supplementary Fig. 2). Notably, Policaptil Gel Retard completely blocked induction of these genes at ZT12 (Supplementary Fig. 2), highlighting a beneficial effect of the tested product on hepatic metabolism under HFD regimen.

The 412 genes specifically upregulated in Policaptil Gel Retard treated animals, were enriched for terms mainly associated with glucose homeostasis, insulin signaling and circadian rhythms (Fig. 2d). Moreover, TF enrichment analysis identified the circadian transcription factor CLOCK as one of the major regulators of these genes (Fig. 2e). Given that HFD causes loss of oscillation of insulin signaling as well as clock regulated metabolic pathways^15^, these results show that Policaptil Gel Retard is able to blunt HFD driven circadian transcriptional reprogramming.

**Figure 3:**
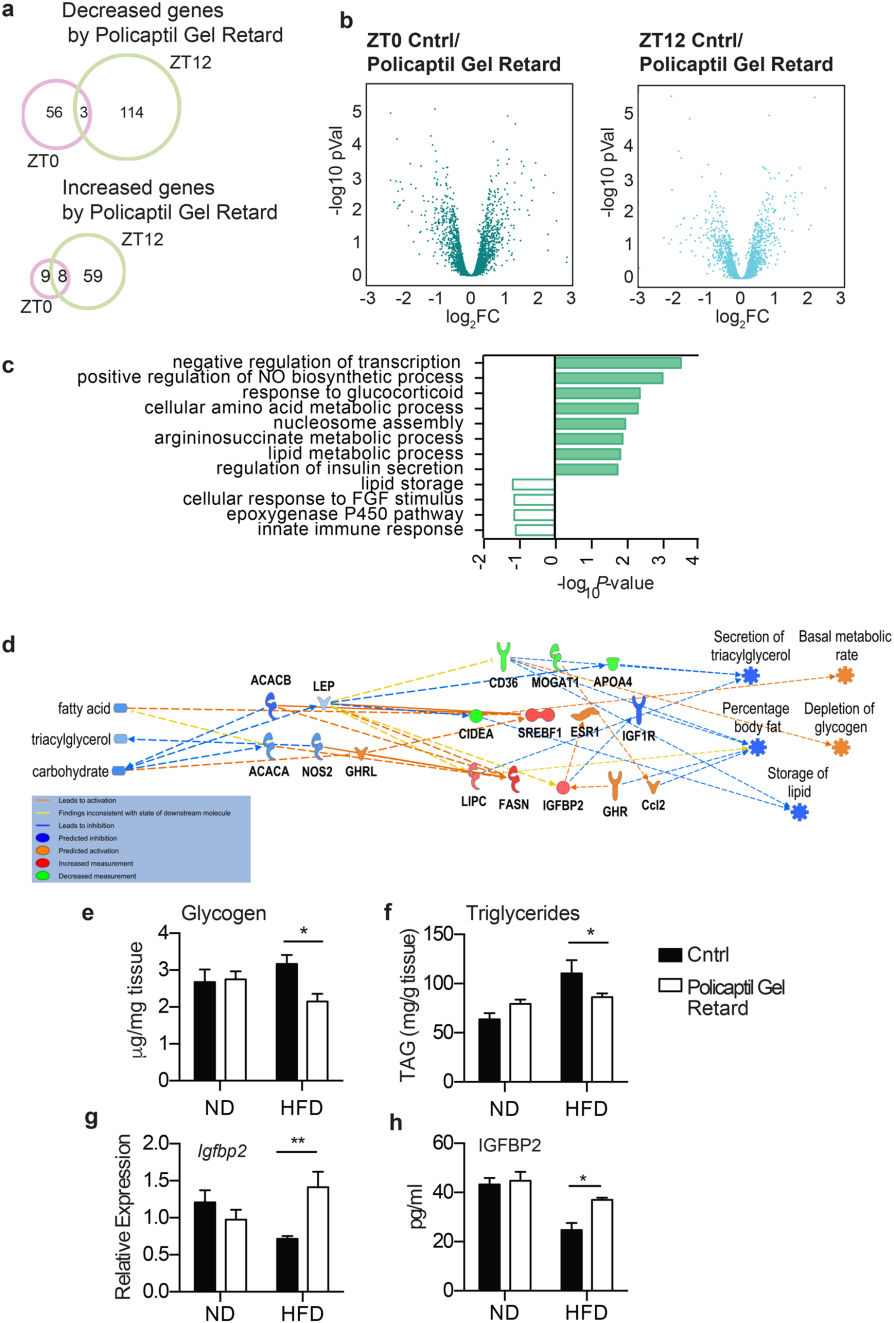
Policaptil Gel Retard affects hepatic energy storage. (**a**) Venn diagram of genes significantly up-regulated or down-regulatd by Policaptil Gel Retard treatment at ZT12 and ZT0 (*Cyber t-test* p < 0.05). (**b**) Volcano plot of differentially expressed genes at ZT0 and ZT12 by Policaptil Gel Retard treatment. (**c**) Pathway enrichment of genes decreased or induced by Policaptil Gel Retard at ZT12. (**d**) In silico modeling with IPA of the systemic effects of Policaptil Gel Retard administration. (**e**) Hepatic glycogen content in response to HFD and Policaptil Gel Retard treatment (mean ± s.e.m, n = 5; *p < 0.05; ANOVA, Holm-Sidak post hoc). (**f**) Hepatic triglycerides content in response to HFD and Policaptil Gel Retard treatment (mean ± s.e.m, n = 5; *p < 0.05; ANOVA, Holm-Sidak post hoc). (**g**) Expression of hepatic Igfbp2 in response to HFD and Policaptil Gel Retard treatment (mean ± s.e.m, n = 5; **p < 0.01; ANOVA, Holm-Sidak post hoc). (**h**) Serum IGFBP2 level in response to HFD and Policaptil Gel Retard treatment (mean ± s.e.m, n = 5; *p < 0.05; ANOVA, Holm-Sidak post hoc).

To further identify genes specifically modulated by Policaptil Gel Retard, we conducted a differential analysis comparing control mice versus animals treated with the tested product. At ZT0 we found 56 genes significantly down-regulated and 9 transcripts up-regulated in Policaptil Gel Retard compared to controls (p < 0.05; Fig. 3a,b). At ZT12 114 transcripts were significantly down-regulated and 59 were up-regulated in mice treated with the tested product compared to controls (p < 0.05; Fig. 3a,b). The greater number of genes modulated at ZT12 compared to ZT0 suggest that Policaptil Gel Retard modulates the hepatic response to nutrients. Functional classification of Policaptil Gel Retard responsive genes at ZT12 highlighted a prominent role in metabolic regulation. Down-regulated genes included genes involved “Lipid storage” indicative of an altered lipid uptake. Conversely, genes induced by tested product were enriched for several metabolic pathways including “response to glucocorticoid”, “lipid metabolic process” and “regulation of insulin secretion” (Fig. 3c). Taken together, these results demonstrate that Policaptil Gel Retard treated mice show changes in the expression of genes involved in glucose and lipid metabolism. In silico modelling of the observed gene expression profiles, allowed us to predict and successfully subsequently validate a depletion of both lipid and glycogen stores in the liver (Fig. 3d). Indeed, both hepatic glycogen and triglyceride content were normalized by administration of Policaptil Gel Retard under HFD regimen, displaying lower levels compared to vehicle treated mice (Fig. 3e,f).

### Policaptil gel retard rescues circulating levels of IGFBP2

Insulin-like growth factor binding proteins (IGFBPs) are major regulators of insulin-like growth factor bioavailability and activity. Therefore, IGFBPs represent an important link between the IGF system and insulin signaling^35^. Hepatic and circulating IGFBP-2 levels are decreased in obese subjects^36–38^. Moreover, Igfbp2 has been shown to protect from the development of insulin resistance in a model of diet-induced obesity^39,40^ and its activity is modulated by the anti-diabetic drug Metformin^41^.

*Igfbp2* was among the genes that exhibited the most significant changes in the liver upon treatment with tested product. Because Policaptil Gel Retard is able to improve insulin sensitivity in mice fed HFD, we postulated that it might exert its effects through the IGFBP2-IGF-1 axis.

We analyzed the expression of *Igfbp2* by qRT-PCR. In the liver of mice fed HFD, *Igfbp2* expression was significantly decreased and its expression was completely rescued by treatment with Policaptil Gel Retard (Fig. 3g). We next investigated if tested product was also able to restore the levels of circulating IGFBP2. Indeed, as observed for the mRNA expression, serum levels of IGFBP-2 were decreased in HFD mice and significantly increased in mice treated with Policaptil Gel Retard (Fig. 3h).

### Policaptil gel retard counteracts modifications of the gut microbiota typically associated with obesity

**Figure 4:**
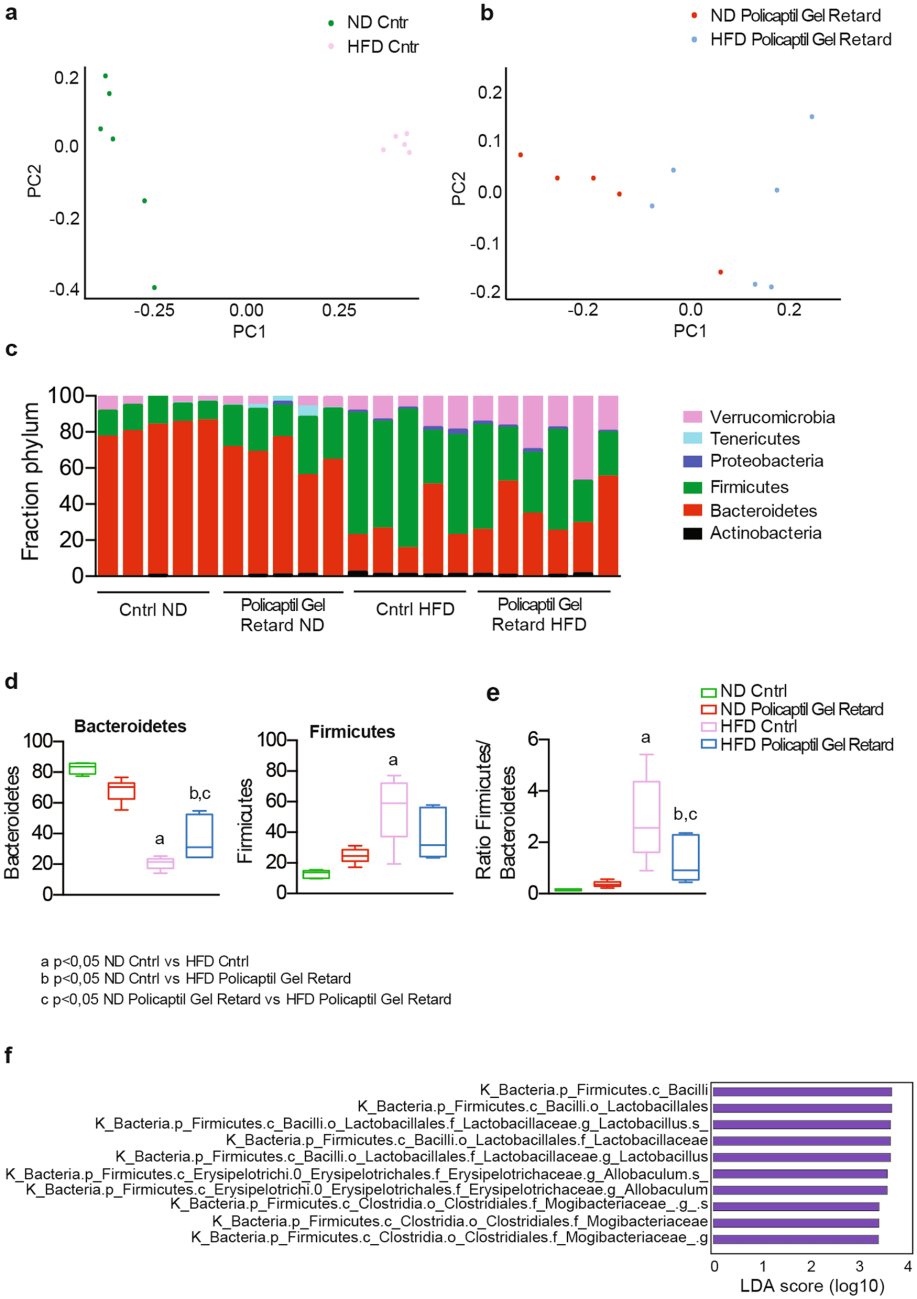
Changes in fecal microbiota induced by Policaptil Gel Retard in mice fed HFD (**a**) Principal coordinate analysis (PCoA) of UniFrac distances of ND and HFD control mice (n = 5–6 mice). (**b**) Principal coordinate analysis (PCoA) of UniFrac distances of ND and HFD Policaptil Gel Retard treated mice (n = 5–6 mice). (**c**) Relative abundance at phylum level in fecal community of control and Policaptil Gel Retard treated mice. (**d**) Representative Phyla Operational Taxonomic Unit (OTU) abundance (%) of ND control, HFD Policaptil Gel Retard, HFD control and HFD Policaptil Gel Retard mice (mean ± s.e.m, n = 5/6; ANOVA, Tukey’s post hoc). (**e**) Firmicutes/Bacteroidetes ratio of ND control, HFD Policaptil Gel Retard, HFD control and HFD Policaptil Gel Retard mice. (mean ± s.e.m, n = 5/6; ANOVA, Tukey’s post hoc) (**f**) LEfSe analysis showing differentially abundant microbiota from HFD cntrl and HFD Policaptil Gel Retard.

The gut microbiota is known to play an important role in energy homeostasis. Furthermore, several studies in mice and humans have reported strong effects of HFD on gut microbiota composition, mainly characterized by enrichment in Firmicutes and depletion of Bacteroidetes^8,42,43^. This shift in microbial composition is associated with an increased potential to extract energy from diet^8^. Changes in gut microbiota composition might contribute to the beneficial effects of Policaptil Gel Retard and are compatible with the liver circadian behavior and serum biochemistry we described. Thus, to clarify the mechanism of action of Policaptil Gel Retard we performed an analysis on the fecal samples of animals treated with the tested product. To this end, fecal microbiota communities were profiled by 16S rRNA sequencing. As expected, PCoA of the unweighted UniFrac distances showed a clear separation between normal chow and HFD in control mice (Fig. 4a). Notably, this shift in microbiota composition was not evident in mice receiving Policaptil Gel Retard (Fig. 4b). These results indicate a possible correlation between the clustering pattern of gut microbiota and the improved metabolic parameters of mice receiving the tested product. In mice fed a ND, Policaptil Gel Retard did not significantly modulate the relative abundances of any of the detected bacteria phyla. However, linear discriminant analysis (LDA) effect size (LEfSe) showed that Policaptil Gel Retard significantly induced the relative abundance of *Clostridiaceae* and *Lachnospiraceae,* which are involved in fiber degradation in the gut and are important for colonic epithelial cells homeostasis and gut health maintenance^44^ (Supplementary Fig. 3).

Remarkably, in the context of HFD Policaptil Gel Retard significantly modulated the relative abundance of several phyla (Fig. 4c). As expected, HFD reduced the abundance of Bacteroidetes and increased the composition of Firmicutes (Fig. 4d). When the tested product was administered, composition of Bacteroidetes marginally increased (Fig. 4d). The effect of Policaptil Gel Retard was more pronounced when looking at the composition of Firmicutes that increased to 57.8% ± 17.7% in the HFD control group and only to 33.5% ± 13.2% in the Policaptil Gel Retard treated group. Bacteroidetes decreased to 26.2 ± 13.8 in the HFD control group and 38.6 ± 13.9in the group treated with the tested group (Fig. 4d). Consequently, the ratio Firmicutes/Bacteroidetes decreased from 2.9 to 1.2 (Fig. 4e). On the other hand, under HFD regimen the Verrucomicrobia phylum was increased in both the control and Policaptil Gel Retard groups (Fig. 4c).

LEfSe was used to identify the most differentially abundant taxa among HFD control and groups treated with the tested product. Members of *Lactobacillaceae*, *Erysipelotrichaceae* and *Mogibacteriaceae* were enriched in HFD control samples and depleted by Policaptil Gel Retard (Fig. 4f). Overall these taxon changes represent a significant reduction in Firmicutes in response to the tested product.

Firmicutes promote the absorption of dietary fats in the gut^45^, leading to an increased energy harvest^8^. Thus, these results indicate that Policaptil Gel Retard is capable of modulating the composition of the gut microbiota in a way that could possibly lead to decreased energy harvest from diet.

### Policaptil gel retard sequestrates nutrients from the diet in the gut lumen

Our results indicate that treatment with Policaptil Gel Retard determines a hepatic transcriptional signature and a composition of the microbiota that is reminiscent of what is observed under caloric restriction, especially in terms of energy harvested from dietary fats^46,47^. This occurs despite the fact that the animals are fed HFD and do not display a reduction in food intake. We therefore investigated whether Policaptil Gel Retard could indeed factually induce a caloric restriction by reducing the availability of dietary components for both the microbiota and absorbing epithelia in the gut.

**Figure 5:**
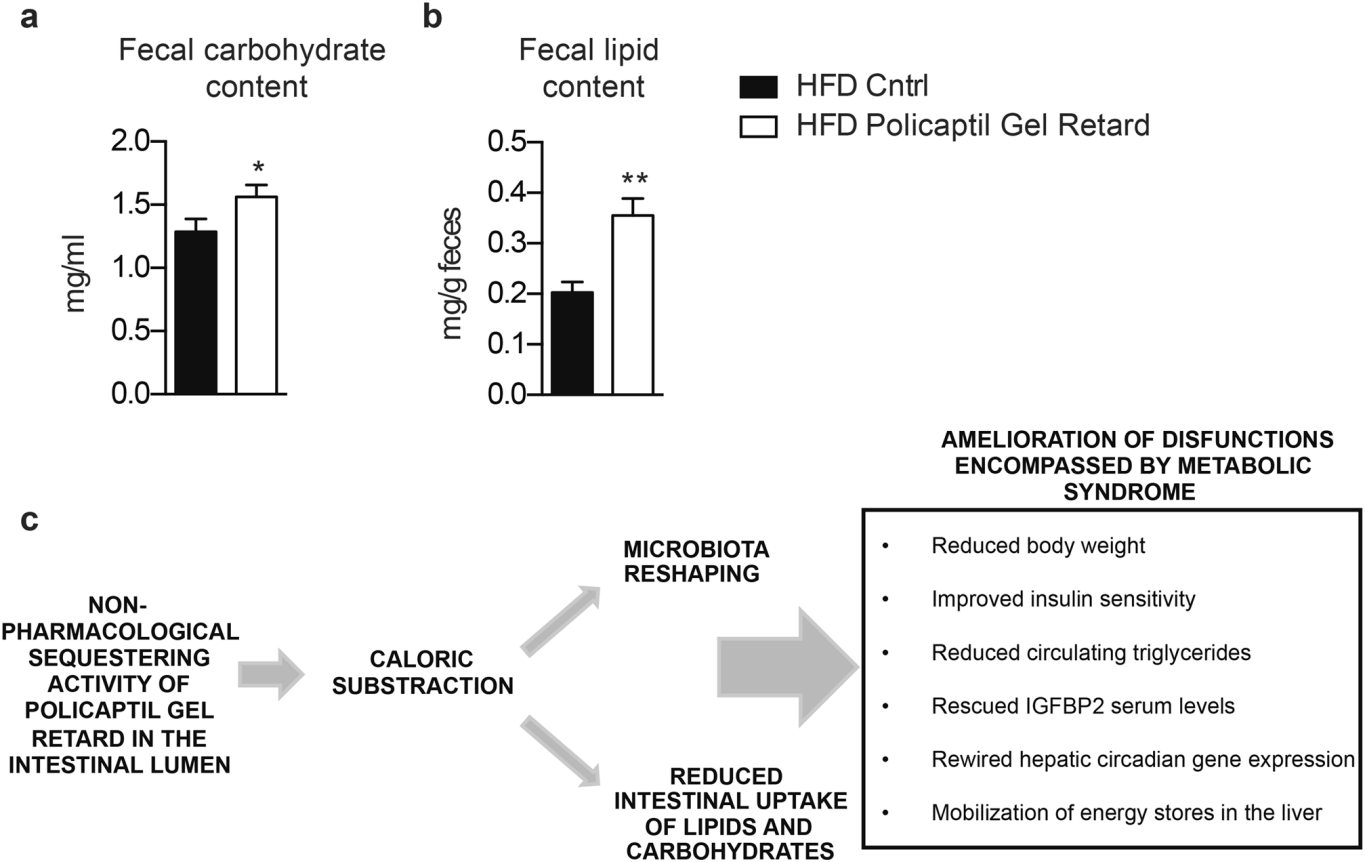
Policaptil Gel Retard reduces gut absorption of lipids and carbohydrates. Fecal carbohydrates (**a**) and lipid (**b**) content level in response to HFD and Policaptil Gel Retard treatment (mean ± s.e.m, n = 6; *p < 0.05, **p < 0.01; unpaired student’s t-test). (**c**) Schematic representation of Policaptil Gel Retard mechanism of action in ameliorating metabolic syndrome phenotypes.

In order to investigate this, we quantified stool lipids and carbohydrates in mice fed HFD and treated with the tested product or vehicle. We observed a significant increase in the amount of lipids and carbohydrates released through the feces already 7 days after Policaptil Gel Retard administration compared to HFD control mice thus confirming that efficacy of the tested product is indeed achieved by reducing absorption of fat and sugar in the gut lumen (Fig. 5a,b).

## Discussion

Here we show the efficacy of a non-pharmacological approach in the management of several metabolic alterations in high-fat diet (HFD) fed mouse model capable of recapitulating aspects of metabolic syndrome. Specifically, we show that treatment with Policaptil Gel Retard results in improved glucose tolerance and insulin sensitivity, rescue of IGFBP2 levels and decrease of serum triglycerides levels. Moreover, we observed a significant reduction of body weight that was not associated with a reduction in food intake, thus highlighting a wide spectrum of beneficial effects in terms of amelioration of parameters defining metabolic syndrome. Importantly, mice fed a normal diet did not display phenotypes in terms of food intake, weight loss, glucose tolerance, insulin resistance, which argues in favor of its safety. Beneficial metabolic effects of Policaptil Gel Retard were coupled with changes in hepatic gene expression. Previous studies have shown that HFD induces gain of oscillation of transcripts with a peak at ZT12, suggesting that ZT12 is the most relevant circadian time point involved in high-fat feeding-dependent transcriptional response^15,32^. Thus, to explore the effects of Policaptil Gel Retard on HFD induced transcriptional reprogramming, we analyzed liver transcriptome at ZT0 and ZT12. In keeping with previous reports, PPARγ, a TF induced in the liver in obesity^48^, was increased at ZT12 in control mice fed HFD and was identified as one of the top represented transcription factors of genes up-regulated at ZT12 in control mice. Notably, PPARγ pathway activation was blunted in the liver of mice treated with Policaptil Gel Retard, indicating that the positive metabolic outcomes of the tested product can be attributed in part to inhibition of ZT specific activation of specific metabolic pathways.

Since Policaptil Gel Retard is designed to exert its effects locally in the gut, its use may be associated to changes in microbiota composition. Indeed, the gut microbiota has extensive effects on systemic metabolism. Gut microbes have been linked to circadian gene expression alterations in the liver of HFD-fed mice^49,50^. Upon high-fat feeding, hepatic diurnal lipid metabolism is the principal metabolic pathway modulated in a microbiota dependent manner^49^. Interestingly, lipid metabolism is also one of the main pathways altered at ZT12 by Policaptil Gel Retard, suggesting that its effects may be mediated by the gut microbiome. Indeed, Policaptil Gel Retard induced alterations in the gut microbial composition that are consistent with an improved metabolic phenotype. Specifically, major changes involved the relative abundance of Firmicutes, coherent with a decrease in energy harvest capability^8^. Upon HFD feeding the gut microbiome is reshaped in a way that favors the uptake and metabolizing of carbohydrates commonly found in western diets^51,52^ and influences hepatic lipid metabolism. Nevertheless, we show that Policaptil Gel Retard increases the amount of carbohydrates secreted in the feces, thereby adding an additional contribution to that coming from reduced lipid absorption and thus potentially further explaining why hepatic metabolism is modulated by treatment with the tested product. Moreover, a number of recent studies have linked the gut microbiota to other tissues involved in the metabolic response to HFD. In fact, the gut microbiota can promote beiging of white adipose tissue (WAT) and thermogenic activation of brown adipose tissue, resulting in enhanced energy expenditure^53,54^. In the present study, we did not investigate the effects of Policaptil Gel Retard on metabolic tissues other than liver, yet it is possible that it may additionally affect metabolism and physiology in other organs.

Taken together, our study identifies the effectiveness of a non-pharmacological approach such as sequestering, thus reducing bioavailability, of dietary components for both the microbiota and the absorbing epithelia in treating the adverse outcomes of diet-induced obesity on dysfunctions by metabolic syndrome. The beneficial outcome of treatment with the tested product can be attributed to two main contributions: on one hand Policaptil Gel Retard modulates the gut microbiome reducing the abundance of energy harvesting species; on the other it prevents hepatic time dependent rewiring of lipid metabolism (Fig. 5c).

## Methods

### Animals, diets and experimental set-ups

Wild type (WT) male C57Bl6 mice were purchased from Jackson Laboratories. Animals were maintained on a 12 h light/12 h dark cycle with ad libitum access to food. Mice were randomly distributed into two groups: untreated (control) and formulated Policaptil Gel Retard-treated (treated) mice. Formulated Policaptil Gel Retard treatment was performed by administering 200 µl of 20 mg/ml of formulated Policaptil Gel Retard in drinking water by oral gavage using a stomach tube. The product solution was prepared fresh just before each treatment. The dose was determined by adjusting the dose recommended for human adults to the relative weight of a medium mouse. Treatment was performed once a day at ZT11 (just before lights are off). Control mice were administered 200 µl of drinking water by oral gavage. Animal care and use was in accordance with guidelines of the Institutional Animal Care and Use Committee at the University of California at Irvine. In the first experimental set-up, mice were subjected to a normal diet (Harlan, 2020X) or high-fat diet (60% kcal fat, Research Diets, D12492) for two weeks ad libitum and treated with formulated Policaptil Gel Retard or vehicle starting from day 0 and throughout the two weeks of experiment. Food intake was measured by weighing the food every 24 h. In the second experimental set-up, after 4 weeks of HFD, mice were randomized in two groups, without statistically significant difference in weight and fasting glycemia, and received either vehicle or formulated Policaptil Gel Retard for 2 weeks. The Institutional Animal Care and Use Committee of the University of California, Irvine approved all experiments (IACUC protocol # AUP-18–22).

### RNA isolation and quantitative PCR

Total RNA was isolated using TRIzol (Invitrogen). 25 mg of liver from control and formulated PolicaptilGel Retard-treated mice were used for RNA preparation. Frozen tissues were resuspended in 1 ml of TRIzol and homogenized using an automated tissue homogenizer. RNA was extracted following the manufacturer´s protocol. RNA concentration was determined using the Nanodrop.

cDNA was prepared by retrotranscription of 1 μg of total RNA using iScript cDNA synthesis kit (Bio-Rad), following manufacturer´s protocol. qRT-PCR was performed by using the real-time CFX96 detection system (Bio-Rad) in a final volume of 20 μl PCR reaction. 3 μl of cDNA was mixed with the specific primers (0.25 μM) and with 10 μl of iQ SYBR Green Supermix (Bio-Rad). The reactions were performed in triplicates using the following conditions: 3 min at 95 °C, followed by 40 cycles of 30 s at 95 °C and 40 s at 60 °C and a final extension step of 5 min at 60ºC.

qRT-PCR data was analyzed using the ∆∆CT method.

### Oral glucose tolerance test (OGTT) and Insulin tolerance test (ITT)

Mice were fasted for 6 h and received glucose (1 g/kg body weight) via oral gavage and insulin (1 U/kg body weight) by intra-peritoneal injection. Blood glucose level was measured using Contour glucose meter (Bayer) prior to injection and 15, 30, 60 and 120 min after glucose or insulin administration.

### Serum biochemistry

For serum collection, blood was left at room temperature for 30 min. Blood clots were then removed by centrifugation at 1,000 *g* for 10 min in a refrigerated centrifuge. The supernatant was collected and IGFBP2 ELISA (Abcam ab207615), Triglyceride assay (Cell Biolabs STA-396) were carried out on samples according to the manufacture’s protocol. Absorbance was measured with Synergy H4 instrument (Biotek).

### Liver measurements

Hepatic lipids were extracted using the Lipid Extraction kit (Cell Biolabs STA-612). Extracted lipids were air dried, resuspended in butanol, and triglycerides were quantified using a Triglyceride quantification kit (Cell Biolabs STA-396) according to the manufacture’s protocol. Hepatic glycogen quantification was measured using the Glycogen Assay Kit (Cell Biolabs STA-5022) according to the manufacture’s protocol.

### Fecal measurements

Fresh fecal pellets were collected and weighed. Fecal lipids were extracted using a Lipid Extraction Kit (Cell Biolabs STA-162). Extracted lipids were air dried, resuspended in butanol, and quantified using a Lipid Quantification Kit for neutral lipids (Cell BiolabsSTA-617). Fecal carbohydrates were quantified using a Total Carbohydrate Assay Kit (Abcam, ab155891) according to the manufacture’s protocol.

### RNA sequencing

RNA sequencing was performed at the UCI Genomics High throughput Facility (https://ghtf.biochem.uci.edu) as previously described following ENCODE guidelines^55^. Total RNA was extracted from liver using Trizol reagent (Gibco BRL Life Technologies, Rockville, MD) and RNA was cleaned with Qiagen RNeasy kit (Qiagen, Chatsworth, CA). Total RNA was monitored for quality control using the Agilent Bioanalyzer Nano RNA chip and Nanodrop absorbance ratios for 260/280 nm and 260/230 nm. Library construction was performed according to the IlluminaTruSeq mRNA stranded protocol. The input quantity for total RNA was 0.75 μg and mRNA was enriched using oligo dT magnetic beads. The enriched mRNA was chemically fragmented for four minutes. First strand synthesis used random primers and reverse transcriptase to make cDNA. After second strand synthesis the ds cDNA was cleaned using AMPure XP beads and the cDNA was end repaired and then the 3′ ends were adenylated. Illumina barcoded adapters were ligated on the ends and the adapter ligated fragments were enriched by nine cycles of PCR. The resulting libraries were validated by qPCR and sized by Agilent Bioanalyzer DNA high sensitivity chip. The concentrations for the libraries were normalized and then multiplexed together. The concentration used for clustering the flowcell was 200 pM. The multiplexed libraries were sequenced on eight lanes using single end 100 cycles chemistry for the HiSeq 4,000. The version of HiSeq control software was HCS 3.3.76 with real time analysis software, RTA.

### Alignment and expression normalization

Sequencing data for 72 samples in FastQ format was produced using post-processing from Illumina software CASAVA 1.8.2 by the Genomics High-Throughput Facility at the University of California, Irvine. Reads failing Illumina's standard quality tests were not included in these FastQ files. The sequencing reads from each sample in the experiment were separately aligned to the reference genome mm10 and corresponding known splice junctions extracted from the UCSC Genome Browser (https://genome.ucsc.edu/) ^56,57^ using the short-read aligner ELAND v2e (Illumina). Reads aligned with a non-unique best match or with two or more mismatches with the reference sequences were discarded from the analyses. The remaining uniquely aligned reads were used to estimate relative transcript abundance for further analysis. Reads per Kilobase of transcript per Million mapped reads (RPKM) were used to quantify the relative abundance of each transcript in a sample and to perform gene expression analyses.

### 16S rRNA sequencing

Gene targeted sequencing was performed on feces collected at ZT12 from mice fed ad libitum treated or not with formulated Policaptil Gel Retard for 11 consecutive days. Fecal DNA was extracted using the QIAamp DNA stool Mini Kit (QIAGEN). Sequencing was carried out with the ZymoBIOMICS Service (Zymo Research, Irvine, CA) as previously described^49^. Briefly, The the v3-4 region of the 16S rRNA gene was amplified with the general bacterial 16S primers 341f (CCTACGGGNGGCWGCAG) and 805r (GACTACHVGGGTATCTAATCC) and sequencing library was prepared by following a published protocol^58^. The amplicon libraries were cleaned up with Zymo’s Select-a-Size DNA Clean & Concentrator (> 200 fragments were kept), quantified, normalized and sequenced on IlluminaMiSeq with v2 reagent kit (500 cycles). Raw sequence reads were trimmed with Trimmomatic-0.33^59^. The two paired-end reads in each pair were assembled to construct a complete amplicon sequence with SeqPrep (https://github.com/jstjohn/SeqPrep). Chimeric amplicon sequences were identified and removed with Usearch (v. 6.1) in ref mode against a curated database (https://drive5.com/uchime/rdp_gold.fa). Amplicon sequences smaller than 320 bp were removed. For each sample, 50,000 sequences were randomly sampled to reduce potential bias caused by uneven sampling. Data was analyzed with Qiime 1.8.0. and the GreenGene database (gg_13_8) as reference database. Singleton OTUs were removed. Taxa significantly different among groups were identified by LEfSe^60^ with default settings (p > 0.05 and LDA effect size > 2).

### Tested medical device

Formulated PolicaptilGel Retard tablet 725 mg^20^ is a natural molecular complex of functional constituents from medicinal plants and natural substances, selected on the basis of the emerging behavior they acquire once pooled in order to generate a final system of molecules, capable of displaying enhanced water binding (WBC) and swelling capacity (SC). Functional components are plant extracts suitably processed such as to concentrate the categories of desired substances that fall into the class of dietary fibers and mucilage: cellulose (26.36%, total fibers ≥ 90%) and cellulose originated from Opuntia ficusindica (23.25%, total fibers ≥ 35%); Glucomannan originated from Amorphophallus konjac (35.64%, total fibers ≥ 80%); inulin from Cichorium intybus (10.07%, Fructooligosaccharides ≥ 30% ); mucilage originated from Althaea officinalis (0.66%, total fibers ≥ 7%), Linum usitatissimum (0.66%, total fibers ≥ 25%) and Tilia flowers and bracts (0.66%, total fibers ≥ 10%). It also contains essential oil of sweet orange (0.2%), essential oil of lemon (0.1%) and natural orange flavor (2.2%).

### Data processing and statistical analyses

Data was processed using Microsoft Excel software. Statistical analyses were performed using GraphPad PRISM V. 6.01. Statistical significance was determined by two tailed t-test for two groups and two-way ANOVA for repeated measurements*.* Power analysis on a pilot study was utilized to determine sample size. In silico modelling linking collected data to predicted biological consequences was generated through the use of IPA (QIAGEN Inc.https://www.qiagenbioinformatics.com/products/ingenuity-pathway-analysis) ^61^.

## Supplementary information

Supplementary information

Supplementary data

## Data Availability

The data that support the findings of this study are available upon request from the corresponding author. The RNA-seq data have been deposited in the Gene Expression Omnibus (GEO) with the accession number GSE137456.
