# Supplementary material for: A non-pharmacological therapeutic approach in the gut triggers distal metabolic rewiring capable of ameliorating diet-induced dysfunctions encompassed by metabolic syndrome
Source: Sci Rep. 2020 Jul 31;10:12915. doi: 10.1038/s41598-020-69469-y (PMC7395094; doi:10.1038/s41598-020-69469-y)
Supplement: Supplementary file 1 — Supplementary information [file 41598_2020_69469_MOESM1_ESM.docx]

**A non-pharmacological therapeutic approach in the gut triggers distal metabolic rewiring capable of ameliorating diet-induced dysfunctions encompassed by metabolic syndrome.**

Carolina Magdalen Greco^1^, Stefano Garetto^2^, Emilie Montellier^1^, Yu Liu^3^, Siwei Chen^3^, Pierre Baldi^3^, Paolo Sassone-Corsi^1*^ and Jacopo Lucci^2*^

Figure S1


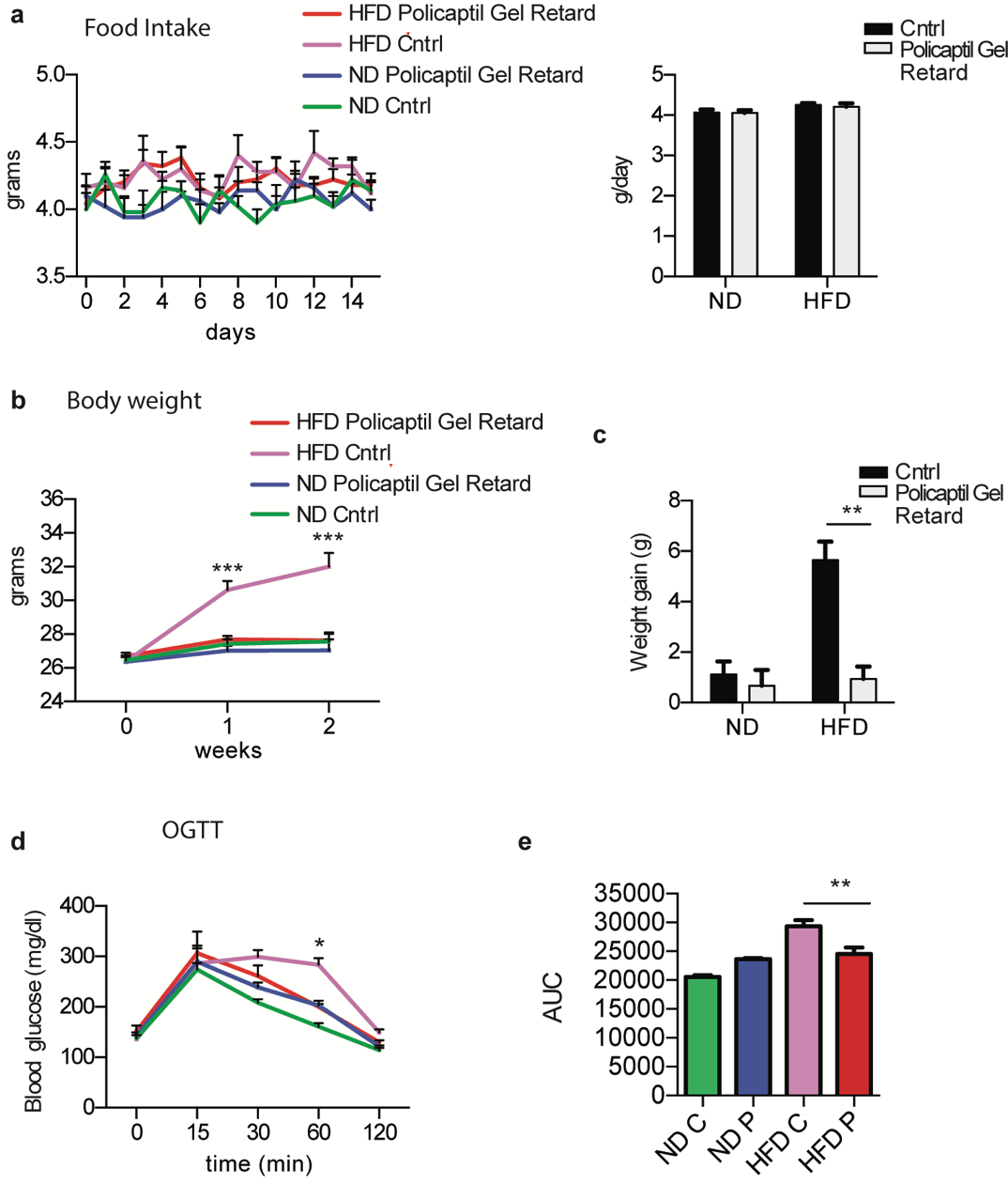


**Supplementary Figure 1: a-left** Daily food consumption of mice treated with vehicle or with Policaptil Gel Retard (mean ± s.e.m, n=5). **a-right** Graph of average food intake of mice treated with vehicle or with Policaptil Gel Retard (mean ± s.e.m, n=5). **b** Body weight of mice fed either ND or HFD and treated with vehicle (ND Cntrl/ HFD Cntrl) or Policaptil Gel Retard (ND Policaptil Gel Retard / HFD Policaptil Gel Retard) for 2 weeks (mean ± s.e.m, n=10; ***p<0.001; ANOVA, Bonferroni post hoc). **c** Graph of grams of weight gained throughout the study (mean ± s.e.m, n=10; **p<0.01; ANOVA, Bonferroni post hoc). **d** Oral glucose tolerance test (OGTT) in mice treated with vehicle or with Policaptil Gel Retard (mean ± s.e.m, n=5; *p<0.05; ANOVA, Bonferroni post hoc). **e** Area under the curve (AUC) (mean ± s.e.m, n=5; **p<0.01; ANOVA, Bonferroni post hoc).

Figure S2


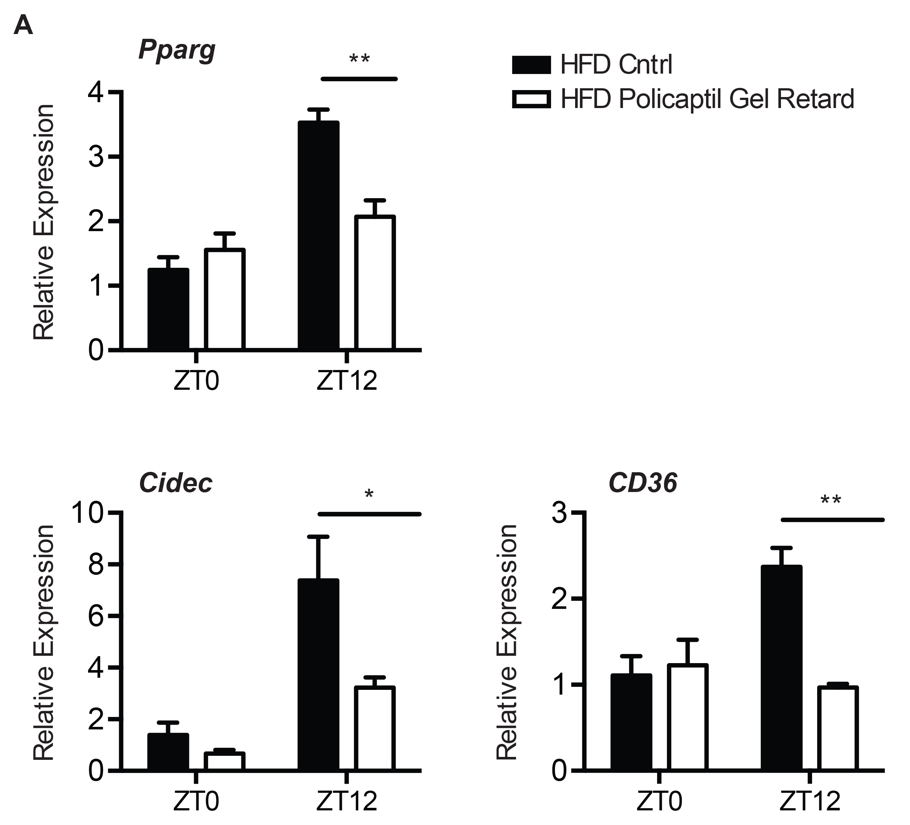


**Supplementary Figure 2: a** Hepatic gene expression of *Pparg* and its target genes *Cidec* and *CD36* in response to HFD and Policaptil Gel Retard treatment (mean ± s.e.m, n=5; *p<0.05, **p<0.01 ; ANOVA, Holm-Sidak post hoc).

**
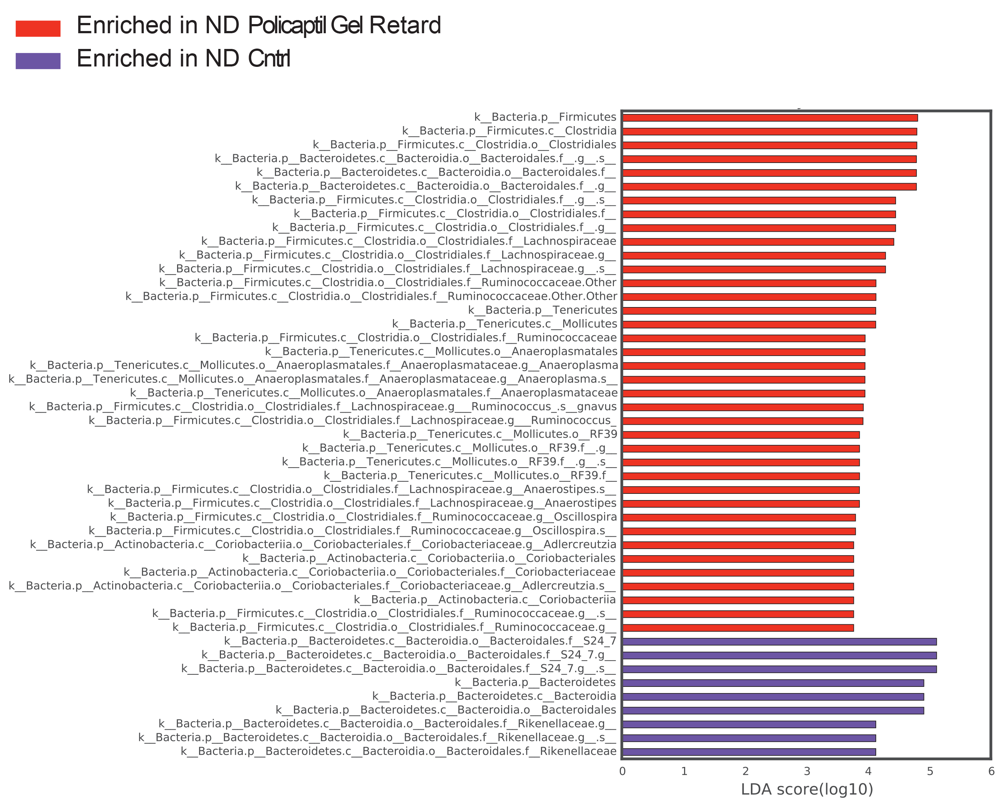
**

**Supplementary Figure 3:** LEfSe analysis showing differentially abundant microbiota from ND cntrl and ND Policaptil Gel Retard .

**Supplementary Table 1:** Comparison between experimental and theoretical WBC and SC of Policaptil Gel Retard (Composition B as of patent nr. EP1679009A1) and its individual components. Theoretical WBC contribution, corresponds to WBC normalized to relative weight of the individual ingredients within Policaptil Gel Retard composition. The data indicates the presence of enhanced WBC and SC values in the final formulation, due to the presence of synergistic effects occurring among individual components.
